# Radiation-induced upregulation of itaconate in macrophages promotes the radioresistance of non-small cell lung cancer by stabilizing NRF2 protein and suppressing immune response

**DOI:** 10.1016/j.redox.2025.103711

**Published:** 2025-06-03

**Authors:** Mengjie Che, Wenwen Wei, Xiao Yang, Jinzi Liang, Yan Li, Ying Ye, Yajie Sun, Yan Hu, Zhanjie Zhang, You Qin, Jing Huang, Bian Wu, Haibo Zhang, Kunyu Yang, Chao Wan, Lu Wen

**Affiliations:** a Cancer Center, Union Hospital, Tongji Medical College, Huazhong University of Science and Technology, Wuhan, 430022, China; b Institute of Radiation Oncology, Union Hospital, Tongji Medical College, Huazhong University of Science and Technology, Wuhan, 430022, China; c Hubei Key Laboratory of Precision Radiation Oncology, Wuhan, 430022, China; d Cancer Center, Department of Radiation Oncology, Zhejiang Provincial People's Hospital (Affiliated People's Hospital), Hangzhou Medical College, Hangzhou, 310000, China

**Keywords:** Radiotherapy, Itaconate, Macrophage, Radioresistance

## Abstract

Radioresistance is one of the important reasons for local recurrence and distant metastasis in non-small cell lung cancer (NSCLC). Itaconate primarily functions as an anti-inflammatory metabolite in macrophages, however, its role in radiotherapy remains to be explored. In this study, we demonstrated that radiation significantly increases itaconate in the tumor microenvironment (TME), which is produced by macrophages. Mechanistically, the NF-κB signaling pathway is rapidly activated in macrophages, which enhances the binding of P65 to the *Acod1* promoter region, leading to significantly increased secretion of itaconate. Excessive itaconate alleviates oxidative stress of NSCLC cell lines by stabilizing NRF2 protein. Notably, specifically knocking out *Acod1* on myeloid cells enhances the activation of the tumor immune microenvironment in response to radiotherapy, particularly increasing the infiltration and activation of CD8^+^ T cells. Therefore, we propose that targeting Acod1 could be an effective strategy to improve radiosensitivity in NSCLC.

## 1 Introduction

Radiotherapy is an effective treatment for patients with early-stage and locally advanced non-small cell lung cancer (NSCLC) who are unable or unwilling to undergo surgery, which can significantly improve the prognosis and quality of life for these patients. In addition, radiotherapy can also significantly improve the survival of patients with advanced NSCLC [[1], [2], [3]]. The primary goal of radiotherapy is to eliminate tumor cells by directly or indirectly damaging their DNA. Furthermore, it can active the immune system to eliminate tumor cells by stimulating an anti-tumor immune response [4]. However, while about 50 % of patients benefit from radiotherapy, radioresistance has emerged as a major factor contributing to local recurrence and distant metastasis. Radioresistance is often due to DNA damage repair, the presence of cancer stem cells, changes in the tumor microenvironment (TME), the role of exosomes, and metabolic reprogramming [5,6]. Studies have indicated that metabolites are crucial in radioresistance [5], yet the specific metabolites require further investigation.

Disruption of the tricarboxylic acid (TCA) cycle and respiratory chain can lead to the accumulation of immune metabolites such as succinate, fumarate, and itaconate, which have a wide diverse range of immunomodulatory functions [7]. In macrophages, *cis*-aconitate decarboxylase (ACOD1), encoded by the immune response gene 1 (*IRG1*), catalyzes the decarboxylation of the tricarboxylic acid TCA cycle intermediate *cis*-aconitate to itaconate [8,9]. Itaconate mainly primarily plays an anti-inflammatory role in macrophages. When during inflammatory stimulation, the expression of Acod1 is rapidly upregulated to mitigate macrophages are stimulated by inflammation, Acod1 will be rapidly up-regulated to reduce the inflammatory response of macrophages [10]. In addition, itaconate has exhibits antibacterial and antiviral effects properties [11,12]. However, the specific role and mechanism of itaconate in radiotherapy remain uncovered.

Our research indicated that itaconate increases rapidly in the TME post-radiotherapy, with Acod1 being upregulated through the activation of the NF-κB pathway, induced by radiation in macrophages. The macrophage-derived itaconate in the TME enhances of tumor cell survival by stabilizing NRF2 protein while suppressing the infiltration and functional activation of CD8^+^ T cells, which contributes to radioresistance of NSCLC. Furthermore, depletion of *Acod1* in macrophages enhanced the radiosensitivity of LLC-bearing mice. In conclusion, targeting radiation-induced itaconate may be a novel strategy to improve the efficacy of radiotherapy in NSCLC.

## 2 Methods

### 2.1 Human specimen collection

Peripheral blood samples were collected from NSCLC patients at the Department of Oncology, Wuhan Union Hospital, Huazhong University of Science and Technology. Each patient provided informed consent prior to blood collection.

### 2.2 Cells lines and cell culture

H1299, LLC, THP-1 cell lines were obtained from the American Tissue Culture Collection (ATCC), H23 cell line was purchased from Procell (Wuhan, China). H1299 and H23 were cultured in RPMI 1640 medium (Gibco, USA) supplemented with 10 % Fetal Bovine Serum (FBS) (#164210-50, Procell, China) and 1 % Penicillin/Streptomycin solution (#BL505A, Biosharp, China). THP-1 was cultured in RPMI 1640 medium supplemented with 10 % FBS, 1 % Penicillin/Streptomycin solution and 0.05 mM β-Mercaptoethanol (Thermo Fisher Scientific, USA). LLC was cultured in DMEM medium (Gibco, USA) supplemented with 10 % FBS and 1 % Penicillin/Streptomycin solution. All cell lines were tested for mycoplasma infection to be negative and cultured in the incubator at 37 °C with 5 % CO2.

### 2.3 Generation of bone marrow-derived macrophages (BMDMs)

Bone marrow cells were collected from the femurs of 6- to 8-week-old female C57BL/6 mice. BMDMs were differentiated in RPMI 1640 medium supplemented with 10 % fetal bovine serum (FBS), 1 % penicillin/streptomycin, and Macrophage Colony-Stimulating Factor (M-CSF) (20 ng/ml, #576406, Biolegend, USA). Red blood cells (RBCs) were removed using an RBC lysis buffer (#BL503B, Biosharp, China). The medium was replaced halfway on the third day and fully replaced on the fifth day. The naïve macrophages were harvested on the sixth day.

### 2.4 Radiation

All cells were irradiated with a single dose of 8 Gy. Mice were anesthetized using a 1 % pentobarbital sodium solution, and the tumor-bearing right lower limbs were irradiated with the same single dose of 8 Gy for three consecutive days. The radiation field dimensions were set to 40 × 40 cm for the cells and 40 × 2 cm for the mice. The radiation parameters included a beam quality of 6 MV and a dose rate of 6 Gy/min, delivered using the Trilogy System Linear Accelerator from Varian Medical Systems.

### 2.5 SiRNA transfection

SiRNAs targeting NRF2 and the negative control (NC) were purchased from RiboBio in Guangzhou, China. SiRNAs targeting RelA, ATF3 and negative control (NC) were obtained from Gene Create in Wuhan, China. Opti-MEM medium was sourced from Gibco in the USA, and Lipofectamine RNAiMAX was acquired from Invitrogen in Carlsbad, CA, USA. Both H1299 and H23 cells were seeded in 6-well plates at a density of 5 × 10^5^ cells per well for transfection. These cells were harvested 48 h later to assess knockdown efficiency and for other treatments. Meanwhile, THP-1 cells were stimulated with Phorbol 12-myristate 13-acetate (PMA) at a concentration of 100 ng/mL (catalog number #ab120297, Abcam) for 24 h before transfection. The sequences of the siRNAs are listed in Supplementary Table S1.

### 2.6 Quantitative real-time polymerase chain reaction (RT-qPCR)

Total RNA was extracted using Trizol reagent (#R401-01, Vazyme, China) and measured in ng/μL using Nanodrop ND-1000 spectrophotometer (Thermo Fisher Scientific, USA). The reverse transcription of RNA to cDNA was performed using HiScript III RT SuperMix (#R323-01, Vazyme, China), and RT-qPCR was performed using AceQ qPCR SYBR Green Master Mix (#Q111-02, Vazyme, China). The sequences of primers are listed in Supplementary Table S2.

### 2.7 Western blotting

Total protein was lysed with RIPA lysis buffer supplemented with protease inhibitors and phosphatase inhibitor at 4 °C for 30 min and centrifuged for 30 min at 12000 g. The supernatant samples were collected and quantified with BCA Protein Assay Kit (#G2026, Servicebio, China), and then denatured at 100 °C for 10 min after adding 5x loading buffer. Protein samples were separated in SDS-PAGE gels and then transferred to PVDF membranes. After blocking with 5 % non-fat milk for 1 h at room temperature, primary antibodies were incubated overnight at 4 °C. The membranes were washed 3 times with TBST for 10 min, incubated with secondary antibodies for 1 h at room temperature, and washed 3 times with TBST for 10 min, followed by chemiluminescent exposure of the blot using NcmECL Ultra (P10100, NCM Biotech). The antibodies mentioned are provided in Supplementary Table S3.

### 2.8 Chromatin immunoprecipitation (ChIP) and ChIP-qPCR assay

Genomic DNA of 200–1000 bp fragments was obtained using the ChIP Assay Kit (#P2078, Beyotime, China) according to the manufacturer's instructions, and then the DNA fragments pulled down by target antibodies was quantified by RT-qPCR. The sequences of primers are listed in Supplementary Table S4.

### 2.9 Transcriptome sequencing (RNA-seq)

Total RNA of BMDMs was extracted using Trizol reagent (#R401-01, Vazyme, China) and sent to NOVOGENE (Beijing, China) for RNA sequencing analysis based on the Illumina platform.

### 2.10 LC-MS/MS analysis

For human blood samples, 200 μL of plasma was taken, followed by 600 μL protein precipitant methanol-acetonitrile (V: V = 2:1), vertexing and shaking for 3 min, incubated at −20 °C for 30 min, centrifuged at 13000 rpm and 4 °C for 10 min, and 500 μL of the supernatant was taken. It was incubated at −20 °C for 30 min, centrifuged at 12000 rpm and 4 °C for 3 min, and 400 μL was taken into the lining tube of the injection bottle for loading analysis. Mouse tumor tissues were collected 24 h after 8 Gy x 3 radiotherapy, then 30 mg of tissue samples were taken and 600 μL of methanol-water (V:V = 4: 1), put in two small steel balls, precool at −20 °C for 2 min, grind (60 Hz, 2 min), shake for 5 min, incubated at −20 °C for 30 min, centrifuged at 13000 rpm, 4 °C for 10 min, and 500 μL of the supernatant was taken. It was incubated at −20 °C for 30 min, centrifuged at 12000 rpm and 4 °C for 3 min, and 400 μL was taken into the lining tube of the injection bottle for loading analysis. After the removal of the protein component, all samples were sent to the GDUT ANALYSIS AND TEST CENTER (Guangzhou, China) for LC-MS/MS analysis.

### 2.11 Clonogenic survival assay

A certain number of cells were seeded in six-well plates and given a specified dose of radiotherapy. After 1–2 weeks, the cell clones were fixed with 4 % paraformaldehyde and stained with crystal violet. Then the clones were photographed and counted, the survival curves were drawn and the radio sensitization enhancement ratio (SER) was calculated using the single-hit multi-target model.

### 2.12 CCK-8 assay

2000 cells were seeded in 96-well plates, given different treatments and cultured for three days. CCK-8 reaction reagent (#C0037, Beyotime) was added according to the manufacturer's instructions, and the cells were incubated at 37 °C for 1 h. The optical absorbance at 450 nm was detected by the microplate reader.

### 2.13 Neutral comet assay

Cells were collected at 4 h and 24 h after 8 Gy irradiation, blown and homogenized into single cell suspension, mixed with low-melting point agarose at 37 °C and the mixture was layered onto preheated slides. Slides were immersed in lysis buffer at 4 °C for 1 h, electrophoresed at 21 V for 45 min in 1x electrophoresis buffer, followed by immersion in DNA precipitation buffer for 30 min, stained with SYBR for 10 min, and visualized using fluorescence microscopy and photographed.

### 2.14 Immunofluorescence staining

For cells, they were fixed with paraformaldehyde and then blocked with 5 %BSA for 1 h, followed by incubation with γH2AX antibody at 4 °C overnight, and incubation with secondary antibody for 1 h. After DAPI staining, cells were observed and photographed using a laser confocal microscope. For tumor tissues and PBMC, they were fixed with paraformaldehyde, embedded in paraffin, sectioned, de-paraffinized and rehydrated, and then performed in 10 mM citrate buffer (pH 6.0) with microwave heating for antigen retrieval. After blocking with donkey serum for 1 h, the slides were incubated with primary antibody at 4 °C overnight and secondary antibody for 1 h. After DAPI staining, the slides were fixed and photographed by a laser confocal microscope. The antibodies mentioned are provided in Supplementary Table S3.

### 2.15 Flow cytometry

For cells, they were digested with trypsin and washed once with PBS, followed by stained with different antibodies according to the manufacturer's instructions, including 7-AAD (#ST515, Beyotime), Annexin V-FITC (#C1062, Beyotime), CM-H2DCFDA (#S0035, Beyotime), C11-BODIPY (#D3861, Invitrogen). For tumor tissues, they were collected and cut into small pieces, digested with collagenase V (#BS954, Biosharp) and hyaluronidase (#BS171, Biosharp) for 1 h at 37 °C, ground on a 40 μm filter, and individual cells were resuspended in PBS after lysis of red blood cells. For the detection of myeloid cells, dead cells were excluded with Zombie NIR™ Fixable Viability Kit (#423106, Biolegend) after blocking the Fc segments, and then stained with CD45 (#157214, Biolegend), CD11b (#101228, Biolegend), F4/80 (#565635, BD Biosciences), CD86 (#105012, Biolegend), CD206 (#141706, Biolegend), Ly6C (#128026, Biolegend) and Ly6G (#127618, Biolegend) antibodies for 30 min at 4 °C to analyze macrophages and MDSCs. For the detection of lymphoid cells, the cells were stimulated with Phorbol 12-myristate 13-acetate (PMA) (100 ng/mL; #ab120297, Abcam), Monensin sodium salt (1 μg/mL; #ab120499, Abcam), and Ionomycin calcium salt (100 ng/mL; #5608212, PeproTech) for 4–6 h. After blocking the Fc segments, the dead cells were excluded with Zombie NIR™ Fixable Viability Kit (#423106, Biolegend), and then stained with CD45 (#103114, Biolegend), CD3 (#100204, Biolegend), CD4 (#100547, Biolegend), CD8 (#752641, BD Biosciences), CD107a (#121626, Biolegend) and NK1.1 (#156504, Biolegend) antibodies at 4 °C for 30 min. After fixation and permeabilization, intracellular staining was performed with IFN-γ (#505829, Biolegend) and FoxP3 (#126408, Biolegend) antibodies at 4 °C for 30 min.

### 2.16 Mice experiments

The procedures on mice experiments were approved by the Ethics Committee of Tongji Medical College, Huazhong University of Science and Technology. C57BL/6J mice and BALB/c nude mice were purchased from Shubeili Biotech (Wuhan, China). C57BL/6J- *Irg1*^em1(IMPC)J/J^ mice (*Acod1*^*−/−*^), Myeloid cell-specific *Acod1*-deficient (*Acod1*^f/f^ *Lyz2*^cre^ mice), were bred and kept in specific-pathogen-free conditions at the Animal Center of Huazhong University of Science and Technology (HUST) in Wuhan, China. The mice used in the experiments were 6–8 weeks sex-matched mice. To establish the subcutaneous transplanted LLC model on C57BL/6J mice, 1 × 10^6^ cells in 100 μL PBS were injected subcutaneously into the right flank of mice, and 5 × 10^6^ cells in 100 μL PBS for the subcutaneous transplanted H1299 model on BALB/c nude mice. When the tumor volume reached 50 mm^3^, mice with similar tumor sizes were randomly divided into different groups. The length(L) and width(W) of tumor was measured every two days and the volume(V) was calculated using formula V = (L × W^2^)/2. Mice were sacrificed if the average diameter exceeded 15 mm or the tumor volumes exceeded 2000 mm^3^.

### 2.17 CD8^+^ T cell depletion

Anti-mouse CD8 monoclonal antibody (clone 2.43) (#A2102, Selleck, China) was intraperitoneally injected at a dose of 200 μg per mouse, and 150 μg per mouse was injected 3 days later. Eyeball blood and spleen of mice were collected the next day to detect clearance efficiency.

### 2.18 Macrophage depletion

Clodronate liposomes (FormuMax, F70101C-AC) at a dose of 200 μL were intraperitoneally injected into mice at the first day, and followed by two doses of 150 μL every two days. Eyeball blood was collected from mice the day after the last injection to detect clearance efficiency.

### 2.19 Statistical analysis

All statistical analysis involved was performed with GraphPad Prism 9.5. The unpaired two-tailed Student's t-test was used for the comparison between two groups, while one-way ANOVA was used for three or more groups. All the graphs show the mean ± SEM. Significant differences between the groups are indicated by ∗: p < 0.05; ∗∗: p < 0.01; ∗∗∗: p < 0.001; and ns, not significant.

## 3 Results

### 3.1 Radiation up-regulates macrophage-derived itaconate

**Fig. 1:**
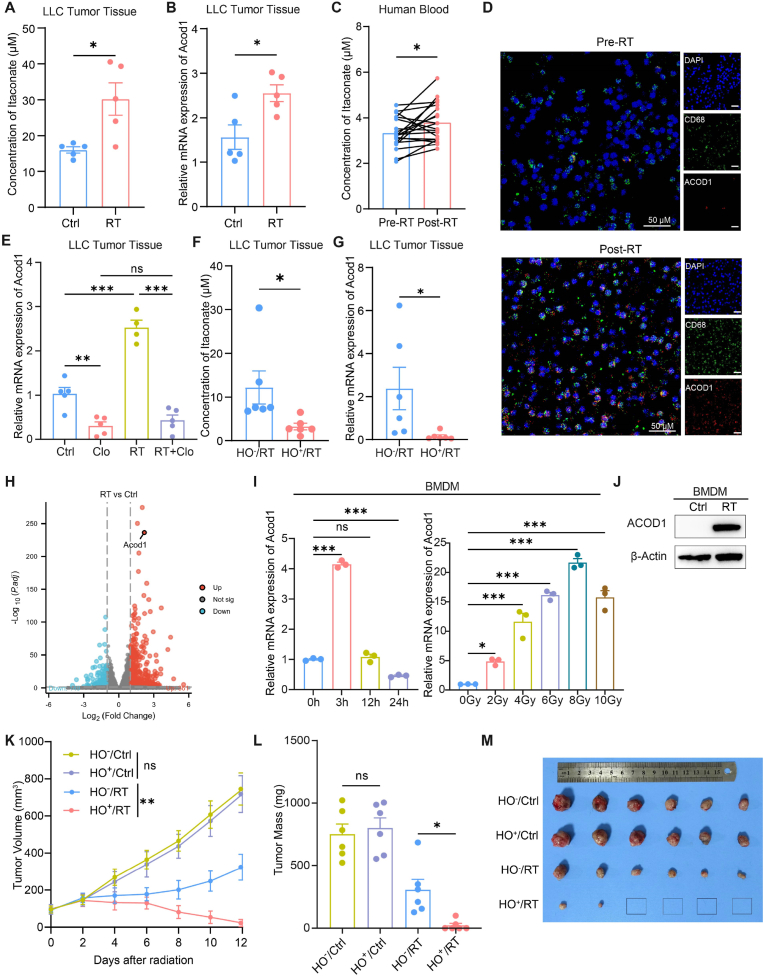
Radiation up-regulates macrophage-derived itaconate **(A)** The concentration of itaconate in LLC subcutaneous tumor tissue grinding fluid before (Ctrl) and after 8 Gy x 3 – radiation (RT) for 24 h detected by LC-MS/MS (n = 5). **(B)** Relative mRNA expression of *Acod1* in LLC subcutaneous tumor tissue before (Ctrl) and after 8 Gy x 3 – radiation (RT) for 24 h (n = 5). **(C)** The concentration of itaconate in paired peripheral blood plasma of NSCLC patients before (Pre-RT) and after radiotherapy (Post-RT) detected by LC-MS/MS (n = 20). **(D)** Immunofluorescence staining of ACOD1 and CD68 expression of paired PBMC samples from lung cancer patients before (Pre-RT) and after radiotherapy (Post-RT). Scale bar = 50 μM. **(E)** Relative mRNA expression of *Acod1* in LLC subcutaneous tumor tissues treated with clodronate liposome (Clo) and/or 8 Gy x 3-radiation (RT) for 24 h (n = 4 or 5). **(F)** The concentration of itaconate in LLC subcutaneous tumor tissue grinding fluid between *Acod1*^*f/f*^*Lyz2*^*cre-*^ (HO^−^)/RT and *Acod1*^*f/f*^*Lyz2*^*cre+*^ (HO^+^)/RT after 8 Gy x 3-radiation for 24 h (n = 6). **(G)** Relative mRNA expression of *Acod1* in LLC subcutaneous tumor tissue between *Acod1*^*f/f*^*Lyz2*^*cre--*^ (HO^−^)/RT and *Acod1*^*f/f*^*Lyz2*^*cre+*^ (HO^+^)/RT after 8 Gy x 3-radiation for 24 h (n = 6). **(H)** Volcano plot of BMDMs after an 8 Gy-radiation for 3 h. **(I)** Relative mRNA expression of *Acod1* in BMDMs at different time points after 8 Gy-radiation and after different doses of radiation for 3 h. **(J)** Representative expression of ACOD1 in BMDMs after an 8 Gy-radiation for 24 h detected by Western blot. **(K)** Tumor growth curves of LLC subcutaneous tumor in different groups (n = 6). **(L)** Tumor weight on day 12 after treatment in different groups (n = 6). **(M)** Image of dissected tumors from each group. ∗p < 0.05; ∗∗p < 0.01; ∗∗∗p < 0.001; ns, not statistically significant.

To investigate the impact of radiotherapy on the secretion of itaconate in the TME, a subcutaneous tumor model of LLC was established in C57BL/6J mice. Tumor tissues were collected at 24 h after radiotherapy (8 Gy each time for three consecutive days, 8 Gy x 3), and the concentration of itaconate in the tissue grinding fluid was measured by liquid chromatography-tandem mass spectrometry (LC-MS/MS). The results showed that itaconate was significantly up-regulated after radiotherapy compared to the control group (Fig. 1A). Additionally, the mRNA expression level of *Acod1* in tumor tissues was detected by PCR, and the results were consistent with itaconate (Fig. 1B). Furthermore, peripheral blood samples were collected from 20 pairs of NSCLC patients before and after radiotherapy, plasma and PBMC were isolated, and the concentration of itaconate in paired peripheral blood plasma was detected by LC-MS/MS. We found that itaconate was significantly increased in the blood after radiotherapy (Fig. 1C). Michelucci et al. demonstrated that under basal conditions, itaconate levels are similar between human and mouse macrophages. However, upon LPS stimulation, human macrophages produce approximately 60 μM of itaconate, whereas mouse macrophages generate around 8 mM—a roughly 100-fold difference [13]. To determine whether radiation-induced macrophages differ in itaconate production between human and mice, we detected the absolute concentrations of itaconate in bone marrow-derived macrophages (BMDMs) and THP-1-derived human macrophages under identical radiation conditions. At basal levels, itaconate concentrations were comparable between human and mouse macrophages. Moreover, radiation did not significantly alter the degree of itaconate upregulation in either species (Fig. S1 A). These findings suggest that under radiation-induced conditions, human macrophages can produce itaconate at levels similar to those observed in mice, potentially mediating comparable biological effects as seen in murine models.

Since itaconate is mainly derived from myeloid cells, especially activated macrophages [14,15], we detected the expression of ACOD1 and CD68 in the paired PBMC using immunofluorescence staining assays Similarly, we observed that ACOD1 expression was upregulated after radiotherapy, mainly expressed in macrophages (Fig. 1D).

To verify whether radiation-induced itaconate is derived from macrophages, we established a subcutaneous LLC tumor model in C57BL/6J mice and depleted macrophages using clodronate liposome (Clo). The clearance efficiency of macrophages in the peripheral blood of mice was over 90 % (Fig. S1 B). We found that macrophage depletion significantly reversed the radiation-induced upregulation of *Acod1*, indicating that macrophage play a major role in the radiation-induced increase of itaconate (Fig. 1E). Next, we used *Acod1*^*f/f*^ *Lyz2*^*cre+*^ (HO^+^) mice, in which *Acod1* was knockout in macrophages, and the control *Acod1*^*f/f*^ *Lyz2*^*cre-*^ (HO^−^) mice. LLC-bearing mice received radiotherapy with 8 Gy x 3 when the tumor tissue reached about 150 mm^3^, and tumor tissues were collected after 24 h. Itaconate levels in the tumor tissues were measured by LC-MS/MS, and the mRNA expression level of *Acod1* was detected by PCR. The results showed that itaconate and *Acod1* in the *Acod1*^f/f^ *Lyz2*^cre+^ (HO^+^) mice were significantly lower than those in the *Acod1*^f/f^ *Lyz2*^cre−^ (HO^−^) mice (Fig. 1F and G). These findings demonstrate that radiation-induced itaconate is mainly derived from macrophages with the TME.

Meanwhile, RNA sequencing was performed on both control and radiated BMDMs. The results indicated that *Acod1* was significantly upregulated in BMDMs after radiotherapy (Fig. 1H). Moreover, BMDMs were irradiated with a dose of 8 Gy, and RNA was collected at 0, 3, 12, and 24 h post-radiation. The results showed that *Acod1* was rapidly upregulated to the highest level at 3 h after radiation. Besides, different doses of radiation (0, 2, 4, 6, 8, 10 Gy) to BMDMs demonstrated that *Acod1* was upregulated to the highest level with an 8 Gy of radiation (Fig. 1 I). The protein level of ACOD1 was also significantly upregulated in the BMDMs after radiation (Fig. 1J). Furthermore, the THP-1 cell line was induced to differentiate into M0 macrophages using PMA (100 ng/mL) for 24 h, followed by radiation. The findings suggested that *Acod1* expression reached its peak at 48 h post-radiation, particularly with 6 Gy of radiation (Fig. S1 C). We also observed that the protein expression of ACOD1 was significantly upregulated in THP-1 cell lines after radiation (Fig. S1 D).

It has been observed that itaconate is increased in tumor tissues following radiotherapy and is mainly produced by macrophages. To further investigate its role in the response to radiotherapy. We used *Acod1*^*f/f*^ *Lyz2*^*cre-*^ (HO^−^) and *Acod1*^*f/f*^ *Lyz2*^*cre+*^ (HO^+^) mice to establish a subcutaneous LLC tumor model. Radiotherapy (8 Gy x 3) was initiated when the tumor reached about 100 mm^3^. The results suggested that specific knockout of *Acod1* in macrophages significantly sensitized the tumors to radiotherapy. Remarkably, there was a 67 % cure rate observed at day 12 in the *Acod1*^*f/f*^ *Lyz2*^*cre+*^ (HO^+^) mice that received radiotherapy (Fig. 1K–M).

### 3.2 Radiation up-regulates *Acod1* in macrophages via activating the NF-κB pathway

**Fig. 2:**
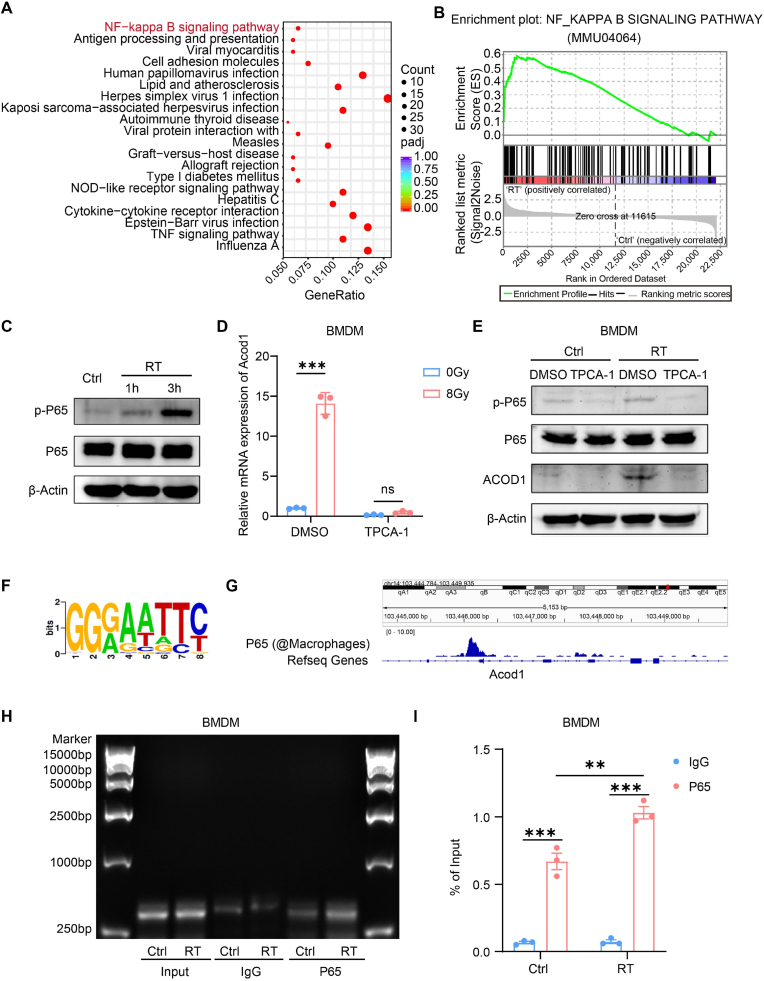
Radiation up-regulates *Acod1* expression in macrophages via activating the NF-κB pathway **(A)** KEGG pathway enrichment in BMDMs after an 8 Gy-radiation for 3 h. **(B)** Gene Set Enrichment Analysis (GSEA) plot and gene sets for NF-kappa B signaling pathway in BMDMs after an 8 Gy-radiation for 3 h. **(C)** Representative Western blot for protein expression levels of P65 and p-P65 in BMDMs after an 8 Gy-radiation for 1 h and 3 h. **(D)** Relative mRNA expression of *Acod1* in BMDMs treated with an 8 Gy-radiation and/or TPCA-1 (1 μM) (NF-κBi). **(E)** Representative expression of P65, p-P65 and ACOD1 in BMDMs treated with an 8 Gy-radiation (RT) and/or TPCA-1 (1 μM) (NF-κBi) detected by Western blot. **(F)** Prediction of P65 binding motifs to *Acod1* promoter regions using ChIPBase v3.0. **(G)** ChIP-seq peak maps of P65 on the promoter region of *Acod1* obtained in the Chip-atlas database. **(H)** Representative image of DNA agarose electrophoresis of the products from the ChIP assay of BMDMs. **(I)** ChIP-qPCR analyses of the products from the ChIP assay of BMDMs. ∗p < 0.05; ∗∗p < 0.01; ∗∗∗p < 0.001; ns, not statistically significant.

To explore the mechanism behind the upregulation of *Acod1* by radiation, we analyzed the transcriptome sequencing of BMDMs before and after 8 Gy-radiation. Enrichment analysis using KEGG and GSEA revealed that the NF-κB signaling pathway was the most significant enriched pathway following radiation (Fig. 2A and B). NF-κB is an essential transcription factor involved in the inflammatory response, and regulates a variety of genes in response to stresses. We detected the expression of P65 and phosphorylated P65 (p-P65) in BMDMs after radiation using Western blot. The results showed that radiation triggers a rapid increase in p-P65 at 3 h post-radiation, while P65 remained unchanged (Fig. 2C). To determine the role of NF-κB in regulating *Acod1* expression, we utilized an NF-κB inhibitor, TPCA-1, which potently inhibits IKK-2. Treatment with TPCA-1 (1 μM) effectively reduced the radiation-induced increase of Acod1 at both mRNA and protein levels in BMDMs (Fig. 2D and E). Giver the radiation quickly causes DNA damage, ATM and ATR complex then rapidly involved in repairing double-strand and single-strand breaks [[16], [17], [18], [19]]. We hypothesized whether the rapid upregulation of *Acod1* induced by radiotherapy might be related to DNA damage repair. We treated control and radiated BMDMs with an ATM inhibitor (KU-55933) (10 μM) and an ATR inhibitor (VE-821) (10 μM). We found that inhibition either ATM or ATR complexes did not reduce the radiation-induced *Acod1* in BMDMs (Fig. S2 A-B). Further verification was performed in the THP-1 cell lines after transfecting siRNA to knock down RelA (P65), the up-regulation of ACOD1 induced by radiotherapy could be reversed (Fig. S2 C-E).

To precisely delineate the upstream pathways regulating radiation-induced *Acod1* expression, we conducted a systematic pharmacological inhibition study targeting three major candidate pathways potentially linking radiation exposure to NF-κB activation and subsequent *Acod1* upregulation.

Radiation-induced cell death is known to release DAMPs, which can activate neighboring macrophages [20]. To investigate the role of DAMP-TLR signaling, we pretreated BMDMs with the MyD88 pathway inhibitor TJ-M2010-5 (10 μM) 8 h prior to 8 Gy irradiation. The results showed that inhibition of the TLR pathway partially reversed the radiation-induced upregulation of *Acod1* mRNA levels, suggesting that DAMPs released after radiotherapy promote *Acod1* expression via TLR signaling (Fig. S2 F). Additionally, since radiation rapidly induces ROS production [21], which can activate NF-κB signaling, we treated BMDMs with the ROS scavenger NAC (5 mM) 8 h before irradiation. ROS depletion also partially reversed the radiation-induced upregulation of *Acod1* mRNA levels (Fig. S2 G), indicating a contribution of ROS-mediated stress to *Acod1* induction. Furthermore, radiation causes DNA damage, leading to the cytosolic accumulation of DNA fragments (e.g., mitochondrial DNA), which can activate the cGAS–STING pathway [22]. While STING classically promotes IRF3-dependent type I interferon responses, recent studies have shown that STING can also activate NF-κB through the TRAF6–TBK1 axis [23]. To test this, we pretreated BMDMs with the STING inhibitor C-176 (5 μM) 8 h before irradiation. Notably, inhibition of the STING pathway completely abolished the radiation-induced *Acod1* upregulation (Fig. S2 H), supporting the hypothesis that radiotherapy may regulate *Acod1* expression via the STING–TRAF6–TBK1–NF-κB signaling axis.

In summary, NF-κB activation following radiotherapy is driven by multiple upstream signals, including ROS accumulation, DAMP–TLR signaling, and activation of the STING pathway. These signals converge to induce *Acod1* expression in macrophages.

Moreover, radiation has been reported to upregulate *Nos2* expression in macrophages, and nitric oxide (NO) can suppress itaconate production by inhibiting mitochondrial respiration and ACOD1 activity [24,25]. To assess whether reducing NO production could influence *Acod1* expression, BMDMs were pretreated with the *Nos2* inhibitor 1400W (50 μM) 8 h before irradiation. The results showed that inhibition of *Nos2* activity partially reversed the radiation-induced upregulation of *Acod1* mRNA levels (Fig. S2 I), suggesting a potential feedback regulation involving NO.

To determine whether NF-κB directly regulates *Acod1* expression in response to radiation, a chromatin immunoprecipitation (ChIP) assay was performed. First, we predicted the binding motif of transcription factor P65 and the *Acod1* gene promoters in BMDMs using ChIPBase v3.0 (Fig. 2F). Additionally, we analyzed the ChIP-seq binding peak map of P65 at the *Acod1* promoter in the Chip-atlas database (Fig. 2G). Our results showed enhanced binding of P65 to the *Acod1* gene promoter in BMDMs after radiotherapy compared to control cells (Fig. 2H and I). This evidence conclusively demonstrates that the NF-κB signaling pathway is involved in the radiotherapy-induced upregulation of *Acod1* expression.

### 3.3 Itaconate reduces the radiosensitivity of NSCLC cells

**Fig. 3:**
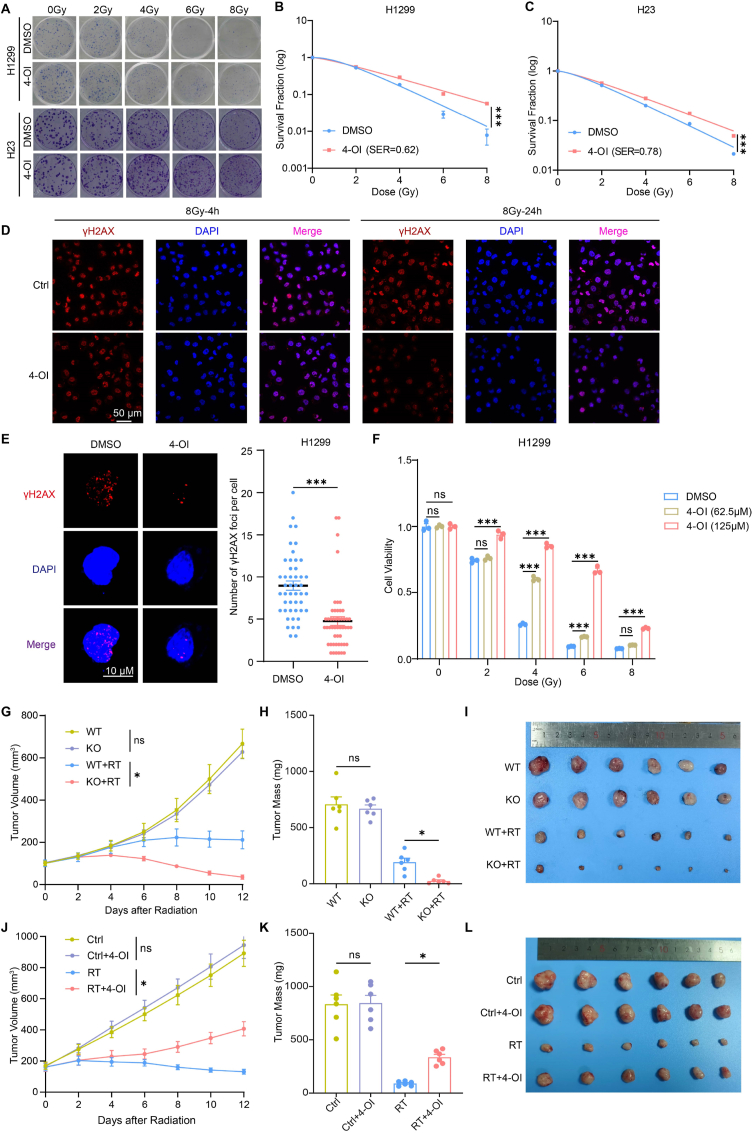
Itaconate reduces the radiosensitivity of NSCLC cells **(A)** Representative images of colony formation assay of H1299 and H23 treated with DMSO or 4-octyl itaconate (4-OI) (125 μM) and irradiated at 0, 2, 4, 6, 8 Gy. **(B–C)** Clonogenic survival assay of H1299 **(B)** and H23 **(C)**. SER, sensitization enhancement ratio. **(D)** Representative confocal images of γ-H2AX foci formation assay of H1299 treated with DMSO or 4-octyl itaconate (4-OI) (125 μM) and collected after an 8 Gy-radiation for 4 h or 24 h. **(E)** Representative and statistical analysis of γ-H2AX foci formation assay of H1299 treated with DMSO or 4-octyl itaconate (4-OI) (125 μM) and collected after an 8 Gy-radiation for 24 h. **(F)** CCK-8 assay of H1299 treated with DMSO or 4-octyl itaconate (4-OI) (62.5 μM, 125 μM) and irradiated at 0, 2, 4, 6, 8 Gy. **(G**–**I)** Tumor growth curves **(G)**, tumor weight **(H)**, and image of tumors **(I)** on day 12 after 8 Gy x 3-radiotherapy of LLC subcutaneous tumor model established in C57BL/6J mice (n = 6). **(J**–**L)** Tumor growth curves **(J)**, tumor weight **(K)** and image of tumors **(L)** on day 12 after 8 Gy x 3-radiotherapy of H1299 subcutaneous tumor model established in BALB/c nude mice (n = 6). ∗p < 0.05; ∗∗p < 0.01; ∗∗∗p < 0.001; ns, not statistically significant.

To investigate the effect of itaconate on the radiosensitivity of cancer cells, we used 4-octyl itaconate (4-OI), which is a cell-permeable derivative of itaconate, to treat tumor cells. We first tested this in two human NSCLC cell lines, H1299 and H23, the clonogenic survival assay showed that exogenous 4-OI (125 μM) significantly reduced the radiosensitivity (Fig. 3A–C). This effect was consistent in LLC, a murine lung cancer cell line (Fig. S3 A-B). Additionally, we measured the number of γH2AX foci to assess the extent of DNA double-strand damage. The results indicated that the number of foci in the H1299 cell line treated with 4-OI did not differ from that of the control group at 4 h post-radiotherapy, but was significantly reduced at 24 h (Fig. 3D and E). Moreover, exogenous addition of 4-OI also significantly decreased DNA damage in the H23 cell line (Fig. S3 C). Meanwhile, we demonstrated that 4-OI inhibits radiation-induced cell death at different doses (Fig. 3F). Radiotherapy induces multiple forms of cell death, including apoptosis, necrosis, and ferroptosis. In addition, it can trigger autophagy—a stress-responsive process that primarily promotes cell survival but may also contribute to cell death under conditions of excessive or prolonged stress [[26], [27], [28], [29]], we found that the cell viability in the presence of 4-OI or inhibitors targeting different pathways was higher than that of the control group (Fig. S3 D). To investigate the pathways involved in itaconate-mediated reduction of cell death, we assessed autophagy, apoptosis, and ferroptosis following 4-OI treatment. The results showed that, after radiotherapy, the levels of all three forms of cell death were significantly reduced in the 4-OI-treated group compared to the control group (Fig. S3 E–I).

To further confirm the radiosensitivity of NSCLC cells *in vivo*, we established a subcutaneous LLC tumor model in C57BL/6J (WT) and *Acod1*^−/−^ (KO)mice. The mice received radiotherapy (8 Gy x 3) when the tumor size reached 100 mm^3^, and were monitored the tumor size. The results showed that *Acod1* knockout significantly enhanced the efficacy of radiotherapy (Fig. 3G–I). Furthermore, to exclude the effect of *Acod1* knockout on immune cells, we established a subcutaneous H1299 tumor model using BALB/c-nude mice. And 25 mg/kg 4-OI was administered daily through continuous intraperitoneal administration. We found that 4-OI inhibits the radiosensitivity of NSCLC in RT group rather than in the control group (Fig. 3J–L). Taken together, our findings suggest that itaconate could reduce the radiosensitivity of NSCLC cells *in vitro* and *in vivo*.

### 3.4 Itaconate induces radioresistance through stabilizing NRF2 protein in NSCLC cells

**Fig. 4:**
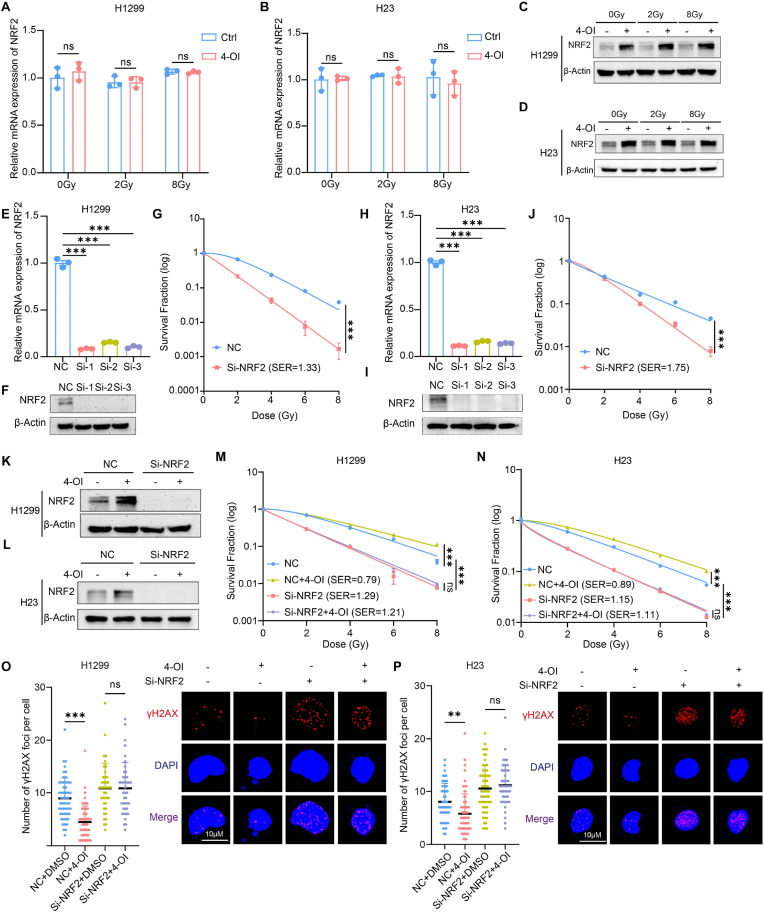
Itaconate induces radioresistance through stabilizing NRF2 protein in NSCLC cells **(A-B)** Relative mRNA expression of *NRF2* in H1299 **(A)** and H23 **(B)** treated with DMSO or 4-octyl itaconate (4-OI) (125 μM) and irradiated at 0, 2, 4, 6, 8 Gy. **(C**–**D)** Representative expression of NRF2 in H1299 **(C)** and H23 **(D)** treated with DMSO or 4-octyl itaconate (4-OI) (125 μM) and irradiated at 0, 2, 4, 6, 8 Gy. **(E**–**J)** mRNA and protein expression level of *NRF2* in H1299 **(E, F)** and H23 **(H, I)** transfected with NC or different siRNAs. Clonogenic survival assay of H1299 **(G)** and H23 **(J)** transfected with NC or si-*NRF2* and irradiated at 0, 2, 4, 6, 8 Gy. **(K**–**L)** Representative expression of NRF2 in H1299 **(K)** and H23 **(L)** transfected with NC or si-*NRF2* and treated with DMSO or 4-octyl itaconate (4-OI) (125 μM) detected by Western blot. **(M**–**N)** Clonogenic survival assay of H1299 **(M)** and H23**(N)** transfected with NC or si-*NRF2* and treated with DMSO or 4-octyl itaconate (4-OI) (125 μM) and irradiated at 0, 2, 4, 6, 8 Gy. **(O–P)** Statistical analysis and representative confocal images of γ-H2AX foci formation assay in H1299 **(O)** and H23 **(P)** transfected with NC or si-*NRF2* and treated with DMSO or 4-octyl itaconate (4-OI) (125 μM) and collected after an 8 Gy-radiation for 24 h ∗p < 0.05; ∗∗p < 0.01; ∗∗∗p < 0.001; ns, not statistically significant.

NRF2 is a key transcription factor that regulates antioxidant stress, and its activation upregulates many antioxidant genes. Itaconate was found to activate NRF2 by alkylating cysteine residues of KEAP1 [30]. Meanwhile, radiotherapy causes an imbalance of redox homeostasis, leading to DNA damage. Therefore, we investigated whether itaconate affects the radiosensitivity of tumor cells by regulating NRF2 expression. Two KEPA1 wild-type human lung cancer cell lines, H1299 and H23, were treated with 4-OI. We found that 4-OI treatment did not induce significantly change the mRNA level of *NRF2* but did significantly upregulate the NRF2 protein level (Fig. 4A–D). Furthermore, we found that antioxidant genes downstream of NRF2 were also up-regulated, and this upregulation remained consistent regardless of the radiation dose (Fig. S4 A-B). Furthermore, a human lung cancer cell line with KEAP1 mutation, H460, was used to explore the effect of KEAP1 in itaconate-induced radioresistance. The results indicated that 4-OI treatment did not alter the radiosensitivity of H460 cells (Fig. S4 C-D). Furthermore, siRNA was transfected into H1299 and H23 cells to knock down *NRF2*, and the knockdown efficiency was assessed by qPCR and Western blot (Fig. 4E–F, H-I). The colony formation assay confirmed that *NRF2* knockdown enhanced the radiosensitivity of tumor cells (Fig. 4G–J). Next, we treated H1299 and H23 with si-*NRF2* and 4-OI, the data showed that exogenous addition of 4-OI did not increase NRF2 expression after *NRF2* knockdown (Fig. 4K and L). However, the colony formation assay revealed that *NRF2* knockdown enhanced the radiosensitivity of tumor cells, while the addition of 4-OI could not reverse this effect (Fig. 4M and N, Fig. S4 E-F). The neutral comet assay and γH2AX foci formation assay also supported these findings (Fig. 4O and P, Fig. S4 G). In conclusion, we demonstrated that itaconate reduces the sensitivity of tumor cells by stabilizing the NRF2 protein, which is dependent on KEAP1.

NRF2 can be activated by both oxidative and electrophilic stress [31], and we hypothesized that itaconate acts as an electrophilic compound that directly modifies cysteine residues on KEAP1, thereby stabilizing NRF2. To test this, H1299 cells were treated under four conditions: DMSO, NAC, 4-OI, and 4-OI + NAC. NAC (5 mM) and 4-OI (125 μM) were administered 6 h prior to 8 Gy irradiation to mitigate ROS and oxidative stress. NRF2 protein levels were significantly elevated in both the 4-OI and 4-OI + NAC groups compared to the DMSO and NAC groups. Importantly, there was no significant difference between the 4-OI and 4-OI + NAC groups, indicating that 4-OI–induced NRF2 stabilization is independent of oxidative stress (Fig. S4 H). These findings support the conclusion that itaconate activates the KEAP1–NRF2 pathway via electrophilic stress.

Beyond NRF2, itaconate has been reported to activate additional immune regulatory pathways, including the ATF3–IκBζ axis and the stimulator of interferon genes (STING) pathway [32,33]. To evaluate whether itaconate influences radiosensitivity through the ATF3–IκBζ pathway, we used siRNA to knock down ATF3 in H1299 cells. Following validation of knockdown efficiency (Fig. S4 I), cells were assigned to four groups: NC + DMSO, NC + 4-OI, siATF3 + DMSO, and siATF3 + 4-OI. Colony formation assays revealed that the number of colonies in the siATF3 + 4-OI group was significantly higher than in the siATF3 + DMSO group after 8 Gy irradiation (Fig. S4 J), indicating that 4-OI does not modulate radiosensitivity through the ATF3–IκBζ axis.

To further investigate whether the STING pathway mediates the effects of itaconate on radiosensitivity, H1299 cells were pretreated with the STING inhibitor C-176 (5 μM) 8 h before irradiation. Cells were divided into four groups: DMSO, 4-OI, C-176, and 4-OI + C-176. Colony formation assays showed no significant difference in colony numbers between the 4-OI + C-176 and 4-OI groups; however, both had significantly more colonies than the C-176 group (Fig. S4 K). These findings suggest that 4-OI does not regulate radiosensitivity through STING-mediated interferon signaling.

### 3.5 Knockout of *Acod1* in macrophages further enhances the activation of tumor immune microenvironment by radiotherapy

**Fig. 5:**
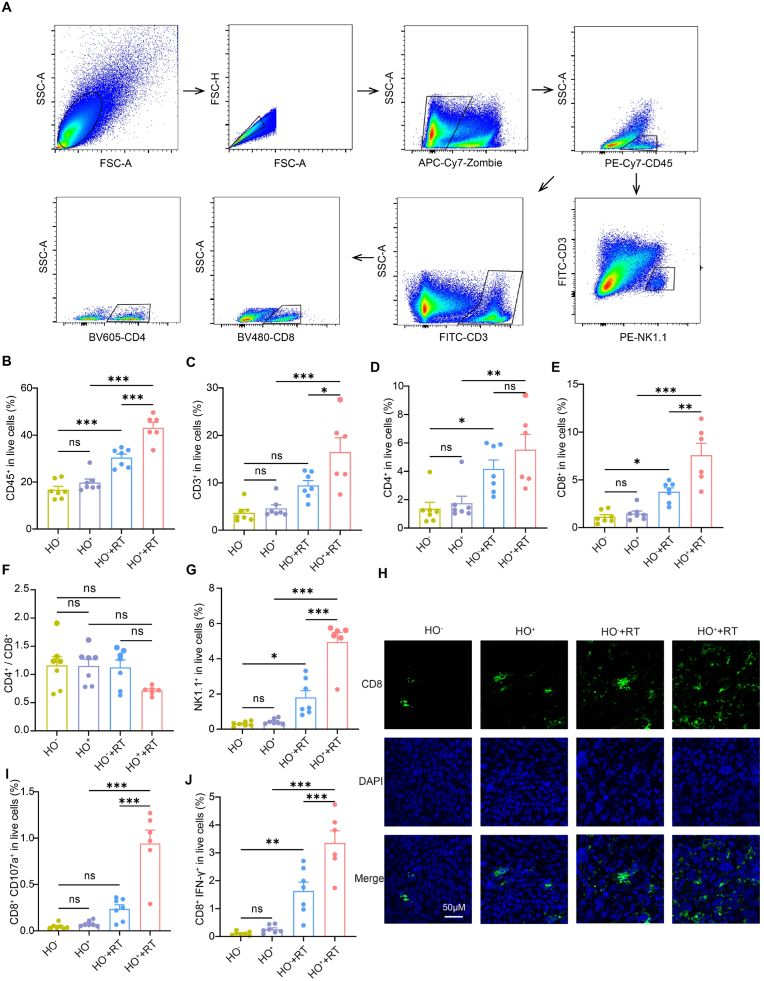
Knockout of *Acod1* in macrophages further enhance the activation of tumor immune microenvironment by radiotherapy **(A)** Gating strategy for detection of NK, CD4^+^ and CD8^+^ T cells by flow cytometry. **(B**–**G)** Flow cytometry analysis of CD45^+^ immune cells **(B)**, CD3^+^**(C)**, CD4^+^**(D)**, CD8^+^**(E)** T cells, the ratio of CD4^+^/CD8^+^ T cells **(F)**, and NK cells **(G)** in the TME of LLC subcutaneous tumor model in mice treated with radiation (8 Gy x 3) in *Acod1*^*f/f*^*Lyz2*^*cre*^^−^ (HO^−^) and *Acod1*^*f/f*^*Lyz2*^*cre+*^ (HO^+^) mice (n = 6). **(H)** Representative immunofluorescence staining of CD8 (green) in the TME at the end of experiment in *Acod1*^*f/f*^*Lyz2*^*cre*^^−^ (HO^−^) and *Acod1*^*f/f*^*Lyz2*^*cre+*^ (HO^+^) mice. **(I**–**J)** Flow cytometry analysis of CD107a^+^**(I)**, and IFN-γ^+^ CD8^+^ T cells **(J)** in the TME of LLC subcutaneous tumor model described above (n = 6). ∗p < 0.05; ∗∗p < 0.01; ∗∗∗p < 0.001; ns, not statistically significant.

As previously mentioned, radiotherapy upregulated the expression of *Acod1* in macrophages, leading to an increase in itaconate in the TME. We demonstrated that itaconate directly affects the radiosensitivity of tumor cells. This prompted us to investigate whether itaconate also impacts the immune cells in the TME after radiotherapy. We constructed a subcutaneous LLC tumor model using *Acod1*^*f/f*^ *Lyz2*^*cre-*^ (HO^−^) and *Acod1*^*f/f*^ *Lyz2*^*cre+*^ (HO^+^) mice. Radiotherapy was administered (8 Gy x3) when the tumor size reached approximately 150 mm^3^. Tumor tissues were collected, and immune cells in the TME were analyzed by flow cytometry at day 7 after the end of radiotherapy (Fig. 5A). The results showed that the proportion of CD45^+^ immune cells (Fig. 5B) and CD3^+^ T cells (Fig. 5C) in the TME was significantly increased in the *Acod1*^*f/f*^ *Lyz2*^*cre+*^ (HO^+^) mice undergoing radiotherapy compared to those receiving radiotherapy alone. Additionally, there was a significant increase in the proportion of CD8^+^ T cells, while the proportion of CD4^+^ T cells did not show a significant difference (Fig. 5D–F). Moreover, the proportion of NK cells was also significantly increased in the *Acod1*^*f/f*^ *Lyz2*^*cre+*^ (HO^+^) mice post-radiotherapy (Fig. 5G). Immunofluorescence analysis of CD8^+^ T cell infiltration in the TME supported our previous findings (Fig. 5H). Further exploring the function of CD8^+^ and CD4^+^ T cells revealed that CD107a^+^ CD8^+^ and IFNγ^+^ CD8^+^ T cells significantly increased in the *Acod1*^*f/f*^ *Lyz2*^*cre+*^ (HO^+^) mice post-radiotherapy compared to those undergoing radiotherapy alone (Fig. 5I and J). This suggests that knockout of *Acod1* in macrophages enhances the activation of CD8^+^ T cells after radiotherapy, while the function of CD4^+^ T cells had no significant change (Fig. S5 B-C).

We also analyzed the proportion of myeloid cells in the TME (Fig. S5 A). Our findings indicated that there is no significant difference in either M1 macrophages or myeloid-derived immunosuppressive cells between HO^−^+RT and HO^+^+RT group (Fig. S5 D-I). This suggests that knockout of *Acod1* in macrophages dose not further enhance the immunosuppressive properties of myeloid cells after radiotherapy.

### 3.6 The antitumor effect of *Acod1* knockout in macrophages combined with radiotherapy partially depends on CD8^+^ T cells

**Fig. 6:**
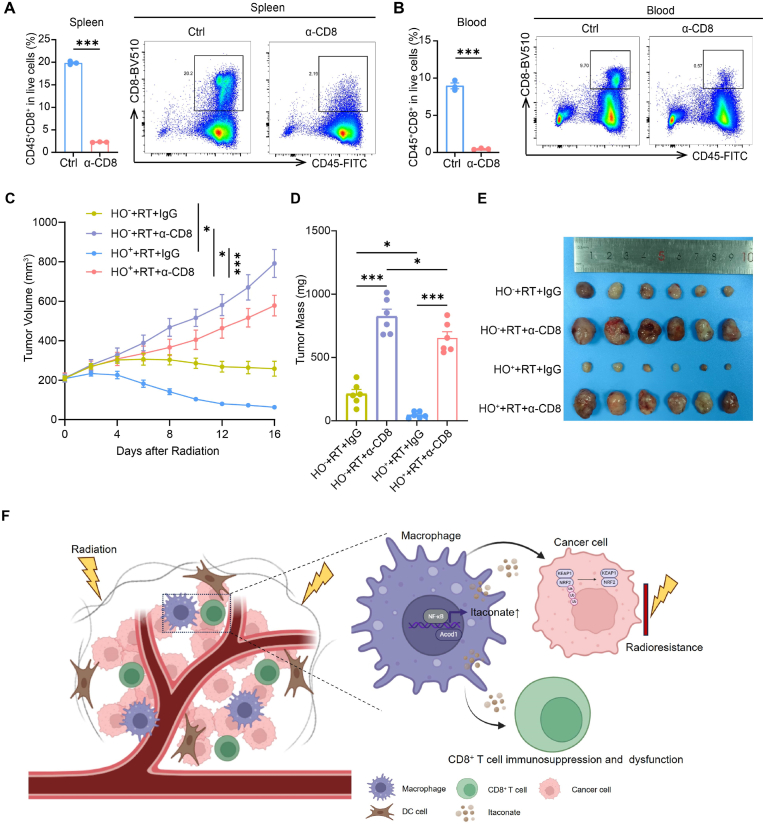
The antitumor effect of *Acod1* knockout in macrophages combined with radiotherapy partially depends on CD8^+^ T cells **(A)** CD8^+^ T cells clearance efficiency in mouse spleen detected by flow cytometry (n = 3). **(B)** CD8^+^ T cells clearance efficiency in mouse peripheral blood detected by flow cytometry (n = 3). **(C)** Tumor growth curves of LLC subcutaneous tumor in *Acod1*^*f/f*^*Lyz2*^*cre*^^−^ (HO^−^) and *Acod1*^*f/f*^*Lyz2*^*cre+*^ (HO^+^) mice treated with 8 Gy x 3 radiotherapy and IgG or neutralizing CD8 antibody (α-CD8) (n = 6). **(D**–**E)** Tumor weight **(D)** and tumor images **(E)** on day 16 after treatment in different groups (n = 6). **(F)** The schematic diagram depicting that macrophages up-regulate the expression of *Acod1* through activating NF-κB pathway after radiotherapy, thereby regulating the radiosensitivity of tumor cells and immune cell infiltration. ∗p < 0.05; ∗∗p < 0.01; ∗∗∗p < 0.001; ns, not statistically significant.

As mentioned above, we discovered that knockout of *Acod1* in macrophages, combined with radiotherapy, further promote the infiltration and activation of CD8^*+*^ T cells. We then investigated the critical role of CD8^+^ T cells in the response to radiotherapy. To do this, we established the LLC subcutaneous tumor model in *Acod1*^f/f^ *Lyz2*^cre−^ (HO^−^) and *Acod1*^f/f^ *Lyz2*^cre+^ (HO^+^) mice, and the neutralizing CD8 antibody was used to deplete CD8^+^ T cells in the mice. The proportion of CD8^+^ T cells in the spleen and peripheral blood was detected by flow cytometry, and the data showed that about 90 % of CD8^+^ T cells were depleted (Fig. 6A and B). The results from *in vivo* experiments showed that both *Acod1*^f/f^ *Lyz2*^cre−^ (HO^−^) and *Acod1*^f/f^ *Lyz2*^cre+^ (HO^+^) mice, when subjected to CD8^+^ T cell depletion, developed significantly larger tumors after radiotherapy compared to the control group. However, the tumor growth in the knockout mice combined with antibody depletion was still slower than that observed in the wild-type mice subjected to the same antibody depletion (Fig. 6C–E). This suggests that the anti-tumor effect of *Acod1* knockout in macrophages combined with radiotherapy is partly dependent on CD8^+^ T cells. Additionally, some of the effect may be a direct effect on the radiosensitivity of tumor cells, as we demonstrated previously.

## 4 Discussion

In this study, we observed an increase in itaconate levels in the TME following radiotherapy. Radiation-induced itaconate production in TAMs was mediated by the upregulation of *Acod1* through the NF-κB pathway. During radiotherapy, itaconate upregulated the expression of downstream antioxidant molecules by stabilizing NRF2, thereby promoting the radioresistance of cancer cells. In addition, itaconate in the TME suppressed the infiltration and activation of CD8^+^ T cells, thereby inhibiting the immunological effects of radiotherapy. Genetic depletion of *Acod1* in mice enhanced T cell-mediated anti-tumor immune responses post-radiotherapy and sensitized the subcutaneous xenograft to radiotherapy. Therefore, targeting itaconate derived from macrophages provided new perspectives for improving the efficacy of radiotherapy in NSCLC (Fig. 6F).

Radiotherapy eliminates cancer cells by inducing DNA damage. However, the metabolism of cancer cells and the TME were altered by ionizing radiation, which might exert both tumor-promoting and tumor-inhibiting effects. On the one hand, radiotherapy profoundly affects ferroptosis-associated lipid metabolism, leading to significant increase in various lysophospholipids (LysoPLs) and diacylglycerols (DAGs) following radiation exposure [34]. Additionally, radiation has been shown to downregulate the expression of SLC7A11 in cancer cells in an ATM-dependent manner, leading to a reduction of cystine uptake and GSH synthesis, ultimately inducing ferroptosis in cancer cells [35]. On the other hand, elevated levels of ROS in the TME after radiation promotes a protumor phenotype in TAMs. This increases the infiltration of Tregs and MDSCs in the TME, which inhibits the anti-tumor function of T cells and NK cells [36]. Furthermore, radiation induces high levels of oxidized lipids in the TME. T cells import these oxidized lipids via CD36, resulting in T cell dysfunction [37]. Therefore, identifying crucial metabolic pathways or key metabolites that significantly influence cancer cells or TME metabolism following radiotherapy could help elucidate the mechanisms of radioresistance and explore strategies to improve the efficacy of radiotherapy. In this study, we found that itaconate, which was derived from macrophage, accumulates in the TME after radiation and is significantly associated with radioresistance. Inhibiting the expression of ACOD1 in macrophages can significantly enhance the radiosensitivity of cancer cells and promote anti-tumor immune responses mediated by T cells.

Radiation is well-known for its immunostimulatory effects, as it enhances antigenicity, promotes the release of damage-associated molecular patterns (DAMPs) and immunostimulatory cytokines, activate the immune system, and increases the presentation of neoantigen [38]. However, the immune suppressive effects of radiation have received limitedly considered. In this study, we found that radiation induces the upregulation of *Acod1* and the release of itaconate in macrophages. Depletion of itaconate enhanced the immune response induced by radiation. Ionizing radiation also upregulates genes like activation of DNA damage, cell cycle arrest, and DNA repair to recover cells from radiation injuries by DNA damage response (DDR) [18]. We suggested that the upregulation of *Acod1* in macrophages after radiation is not induced by DDR, which is not inhibited by ATM and ATR inhibitors. Furthermore, the activation of the NF-κB signaling pathway is modulated by crosstalk with the STING pathway, as well as by diverse upstream mechanisms including ROS-mediated stress and DAMP-triggered TLR signaling. Based on these mechanistic insights, we propose that radiotherapy induces ROS generation, DAMP release, and STING pathway activation, which together converge to activate NF-κB, promotes the phosphorylation of P65, leading to increase the expression of *Acod1*. Itaconate appears to play an immunosuppressive role during radiotherapy, targeting itaconate may potentially enhance the efficacy of radiotherapy by recovering the activation of the immune system induced by radiotherapy.

Wang et al. (2022) reported that 4-OI suppresses ROS accumulation by activiting NRF2-driven antioxidant genes (e.g., *HO-1, NQO1*), thereby protecting melanocytes and keratinocytes from apoptosis. This supports the NRF2-dependent antioxidant role of itaconate derivatives in a different stress context (UVB) [39]. In our study, we demonstrated that itaconate regulates multiple cell death pathways, including apoptosis, via the NRF2 pathway in the context of radiotherapy. Furthermore, we identified both the cellular source of itaconate and the mechanism underlying its upregulation following radiotherapy. Besides, somatic mutations in NRF2 and KEAP1 are commonly observed in NSCLC, which is associated with therapy resistance [[40], [41], [42]]. Our research indicates that itaconate promotes the radioresistance of KEAP1 wide-type lung cancer cells, but does not affect KEAP1 mutated cancer cells *in vitro*. However, itaconate showed a suppressive effect on CD8^+^ T cells in our study. Targeting itaconate may enhance the immune response of radiotherapy, which provides a novel strategy for treating KEAP1 mutated NSCLC.

Myeloid cells, such as peritoneal tissue-resident macrophages (pResMϕ), polymer-phonuclear myeloid-derived suppressor cells (PMN-MDSCs), monocytes, and macrophages, produce itaconate in the TME. This itaconate is taken up by T cells, leading to the suppression of proliferation, recruitment, and function of CD8^+^ T cells, contributing to immune suppression [15,43,44]. In addition, itaconate accumulates in tumor-infiltrating neutrophils (TINs), where it sustains their survival by inhibiting ferroptosis and promoting breast cancer metastasis [45]. A recent study has shown that tumor cells uptake macrophage-derived itaconate through the SLC13A3, which contributes to tumor resistance to ferroptosis [46]. We demonstrated that itaconate upregulates NRF2 expression in cancer cells after radiation, and inhibiting NRF2 expression reverses the radioresistance induced by itaconate.

In summary, our study implies that itaconate significantly decreases the radiosensitivity of NSCLC by enhancing the survival of cancer cells and suppressing CD8^+^ T cells within the tumor microenvironment (TME). Therefore, targeting itaconate may be a promising strategy to enhance the effectiveness of radiotherapy.

## CRediT authorship contribution statement

**Mengjie Che:** Writing – original draft, Investigation. **Wenwen Wei:** Investigation. **Xiao Yang:** Investigation. **Jinzi Liang:** Data curation, Writing – review & editing. **Yan Li:** Resources. **Ying Ye:** Resources. **Yajie Sun:** Resources. **Yan Hu:** Resources. **Zhanjie Zhang:** Supervision. **You Qin:** Supervision. **Jing Huang:** Supervision. **Bian Wu:** Supervision. **Haibo Zhang:** Supervision. **Kunyu Yang:** Writing – review & editing, Methodology. **Chao Wan:** Writing – original draft, Conceptualization. **Lu Wen:** Writing – original draft, Conceptualization.

## Availability of data and materials

The data supporting this study's findings are available from the corresponding authors upon reasonable request.

## Ethics approval statement

Animal experiments were performed under guidelines approved by the Institutional Animal Care and Use Committee of Huazhong University of Science and Technology (IACUC number: 4203). The study received approval from the Ethics Committee of Union Hospital, Tongji Medical College, Huazhong University of Science and Technology (ethical approval number: UHCT-IEC-SOP-016-03-01).

## Funding statement

This work was supported by the 10.13039/501100012166National Key Research and Development Program of China (2016YFC0105311), 10.13039/501100001809National Natural Science Foundation of China (Grant No. 82303715, 82272901, 82373235), the 10.13039/501100002858China Postdoctoral Science Foundation (2023M731218).

## Declaration of competing interest

The authors have declared that no competing interest exists.

## Data Availability

Data will be made available on request.
