# Supplementary material for: Radiation-induced upregulation of itaconate in macrophages promotes the radioresistance of non-small cell lung cancer by stabilizing NRF2 protein and suppressing immune response
Source: Redox Biol. 2025 Jun 3;85:103711. doi: 10.1016/j.redox.2025.103711 (PMC12173628; doi:10.1016/j.redox.2025.103711)
Supplement: Multimedia component 1 [file mmc1.docx]

**Radiation-induced Upregulation of Itaconate in Macrophages Promotes the Radioresistance of Non-Small Cell Lung Cancer by Stabilizing NRF2 protein and Suppressing Immune Response**

Mengjie Che^#a,b,c^, Wenwen Wei^#a,b,c^, Xiao Yang^#a,b,c^, Jinzi Liang^#a,b,c^, Yan Li^a,b,c^, Ying Ye^a,b,c^, Yajie Sun^a,b,c^, Yan Hu^a,b,c^, Zhanjie Zhang^a,b,c^, You Qin^a,b,c^, Jing Huang^a,b,c^, Bian Wu^a,b,c^, Haibo Zhang^d^, Kunyu Yang^a,b,c^, Chao Wan*^a,b,c^, Lu Wen*^a,b,c^

^a^ Cancer Center, Union Hospital, Tongji Medical College, Huazhong University of Science and Technology, Wuhan 430022, China

^b^ Institute of Radiation Oncology, Union Hospital, Tongji Medical College, Huazhong University of Science and Technology, Wuhan 430022, China

^c^ Hubei Key Laboratory of Precision Radiation Oncology, Wuhan 430022, China

^d^ Cancer Center, Department of Radiation Oncology, Zhejiang Provincial People’s Hospital (Affiliated People’s Hospital), Hangzhou Medical College, Hangzhou, 310000, China

^#^ These authors have contributed equally to this article.

* Corresponding authors: E-mail: Lu Wen [wenlu2808@126.com](mailto:wenlu2808@126.com); Chao Wan, [wanc@hust.edu.cn](mailto:wanc@hust.edu.cn)

**Supplementary materials**

**
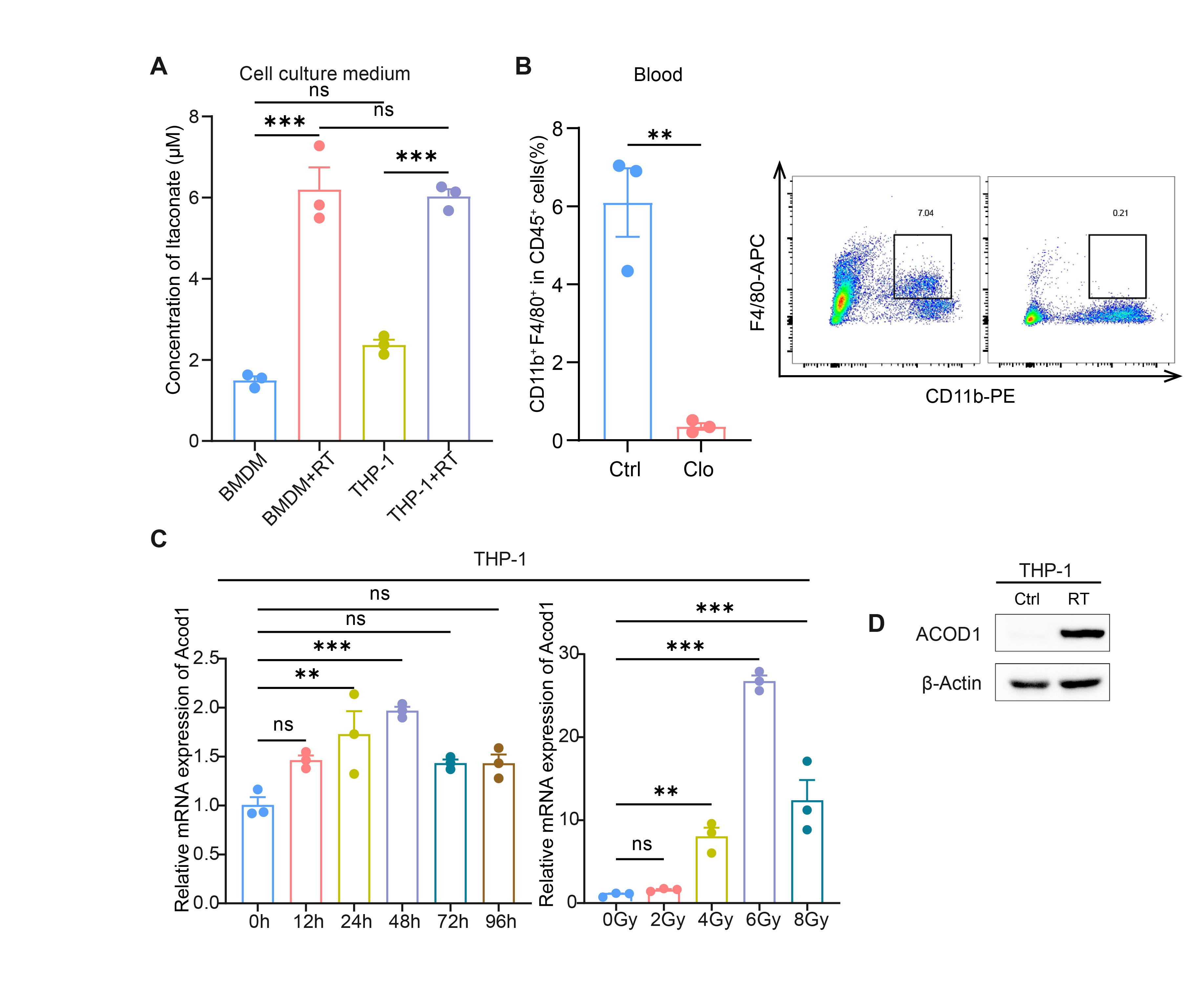

Fig S1: Radiation up-regulates macrophage-derived itaconate**

**(A)** The concentration of itaconate in cell culture medium of BMDM and THP-1 before and after 8 Gy-radiation for 24 h detected by LC-MS/MS (n = 3). **(B)** Macrophage clearance efficiency in mouse peripheral blood detected by flow cytometry (n = 3).  **(C)** Relative mRNA expression of *Acod1* in THP-1 at different time points after 8Gy-radiation and after different doses of radiation for 48 h. **(D)** Representative expression level of ACOD1 in THP-1 after an 8 Gy-radiation for 48 h. *p < 0.05; **p < 0.01; ***p < 0.001; ns, not statistically significant.


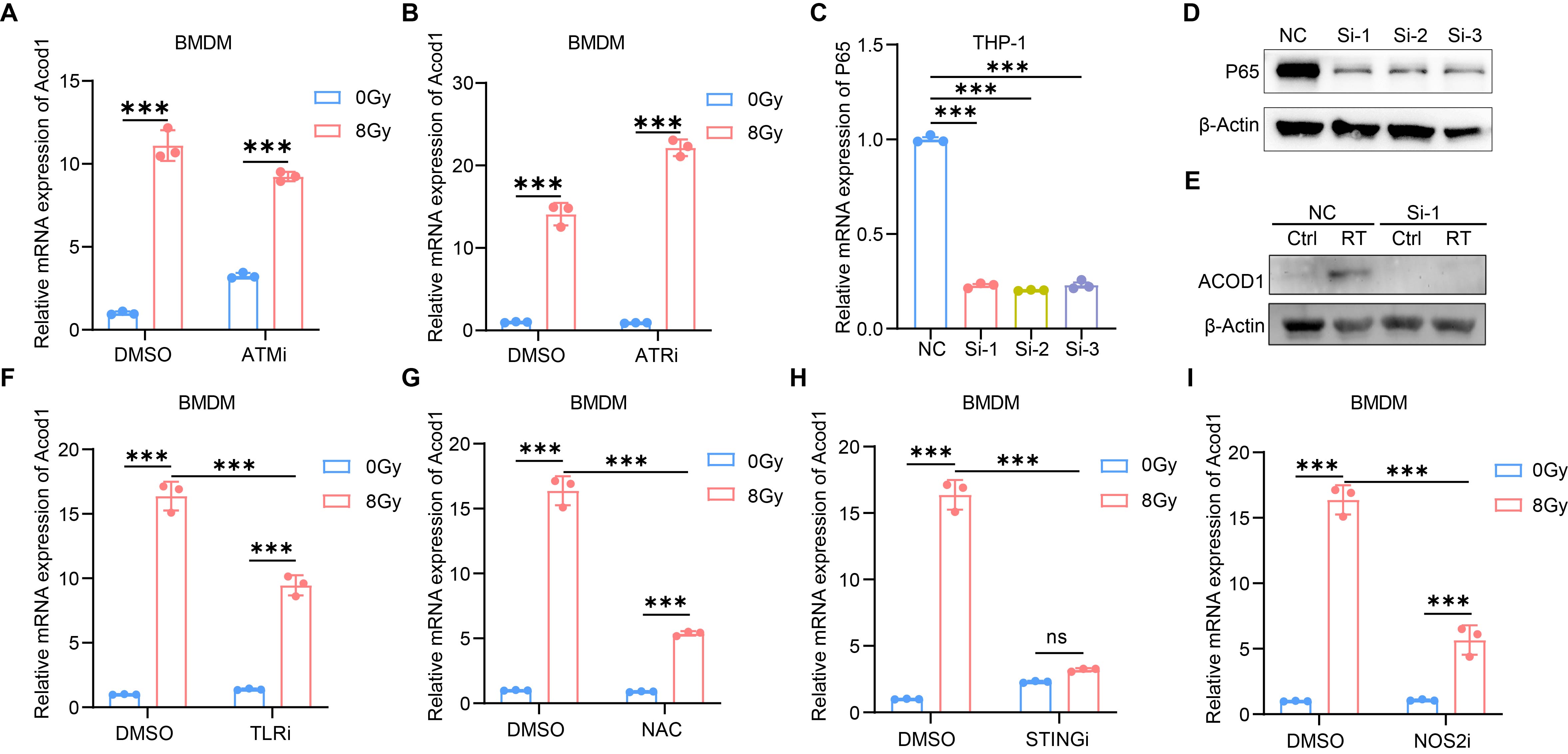


**Fig S2:** **Radiation up-regulates *Acod1* expression in macrophages via activating the NF-κB pathway.**

**(A)** Relative mRNA expression of *Acod1* in BMDMs treated with an 8 Gy-radiation and/or KU-55933 (10 μM) (ATMi). **(B)** Relative mRNA expression of *Acod1* in BMDMs treated with an 8Gy-radiation and/or VE-821 (10 μM) (ATRi). **(C)** Relative mRNA expression of *P65* in THP-1 transfected with NC or siRNAs. **(D)** Representative expression of P65 in THP-1 transfected with NC or siRNA. **(E)** Representative expression of ACOD1 in THP-1 transfected with NC or siRelA after an 8 Gy-radiation for 24 h. **(F)** Relative mRNA expression of *Acod1* in BMDMs treated with an 8 Gy-radiation and/or TJ-M2010-5 (10 μM) (TLRi). **(G)** Relative mRNA expression of *Acod1* in BMDMs treated with an 8 Gy-radiation and/or ROS scavenger NAC (5 mM). **(H)** Relative mRNA expression of *Acod1* in BMDMs treated with an 8 Gy-radiation and/or C-176 (5 μM) (STINGi). **(I)** Relative mRNA expression of *Acod1* in BMDMs treated with an 8 Gy-radiation and/or 1400W (50 μM) (NOS2i). *p < 0.05; **p < 0.01; ***p < 0.001; ns, not statistically significant.

**
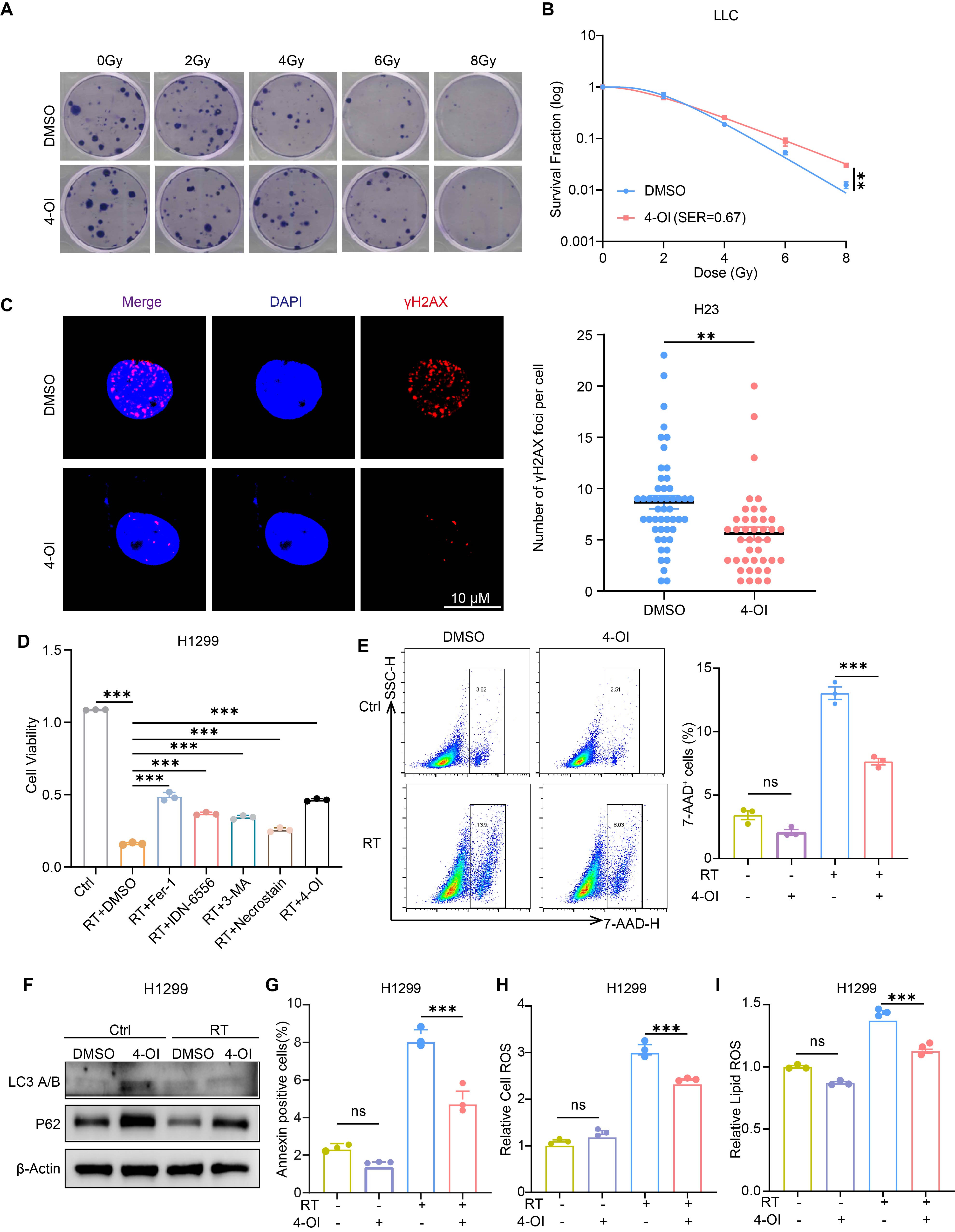
**

**Fig S3:** **Itaconate reduces the radiosensitivity of NSCLC cells**

**(A)** Representative images of colony formation assay of LLC treated with DMSO or 4-octyl itaconate (4-OI) (125 μM) and irradiated at 0, 2, 4, 6, 8 Gy. **(B)** Clonogenic survival assay of **(A)**. SER, sensitization enhancement ratio. **(C)** Representative confocal images and statistical analysis of γ-H2AX foci of H23 treated with DMSO or 4-octyl itaconate (4-OI) (125 μM) and collected after an 8 Gy-radiation for 24 h. **(D)** CCK-8 assay of H1299 treated with various small-molecule cell death inhibitors and an 8 Gy-radiation. **(E)** Representative plots and statistical analysis of 7-AAD-positive H1299 cells treated with an 8 Gy-radiation and/or 4-octyl itaconate (4-OI) (125 μM) detected by flow cytometry. **(F)** Representative expression of LC3 A/B and P62 in H1299 treated with an 8 Gy-radiation and/or 4-octyl itaconate (4-OI) (125 μM) detected by western blot. **(G)** Annexin positive H1299 cells with 125 μM and/or radiation detected by flow cytometry. **(H)** Relative cell ROS measurement (CM-H2DCFDA) of H1299 cells treated with an 8 Gy-radiation and/or 4-octyl itaconate (4-OI) (125 μM) detected by flow cytometry. **(I)** Relative lipid ROS measurement (C11-BODIPY) of H1299 cells treated with an 8 Gy-radiation and/or 4-octyl itaconate (4-OI) (125 μM) detected by flow cytometry. *p < 0.05; **p < 0.01; ***p < 0.001; ns, not statistically significant.


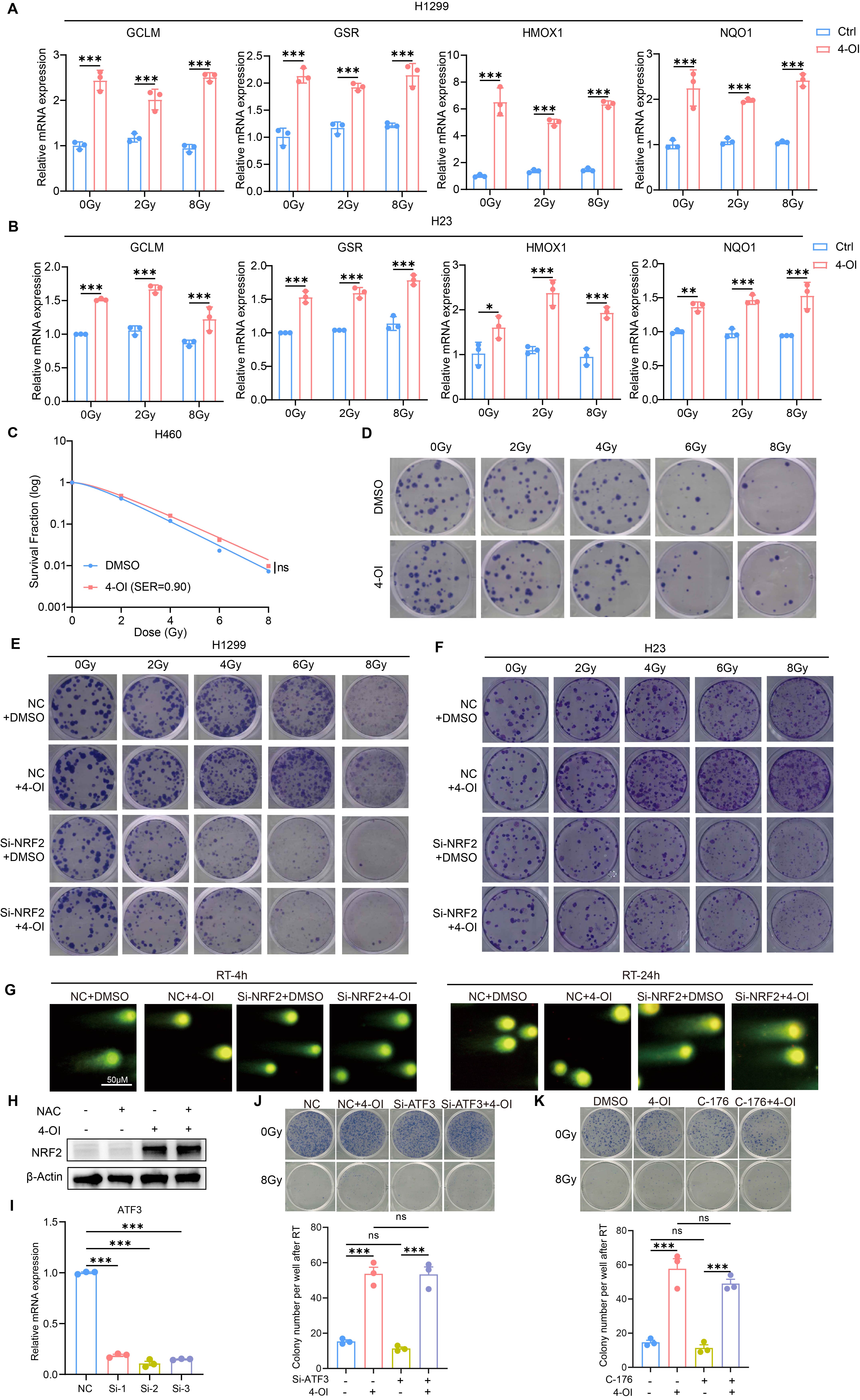


**Fig S4: Itaconate induces radioresistance through stabilizing NRF2 protein in NSCLC cells**

**(A-B)** Relative mRNA expression of antioxidant molecules downstream of *NRF2 (GCLM, GSR, HMOX1, NQO1)* in H1299 **(A)** and H23 **(B)** treated with DMSO or 4-octyl itaconate (4-OI) (125 μM) and irradiated at 0, 2, 8 Gy. **(C-D)** Clonogenic survival **(C)** and representative images of colony formation **(D)** of H460 cells treated with DMSO or 4-octyl itaconate (4-OI) (125 μM) and irradiated at 0, 2, 4, 6, 8 Gy. **(E-F)** Representative images of colony formation assay of H1299 **(E)** and H23 **(F)** transfected with NC or si-NRF2 and treated with 125 μM 4-OI and irradiated at 0, 2, 4, 6, 8 Gy. **(G)** Representative images of neutral comet assay in H1299 transfected with NC or si-NRF2 and treated with DMSO or 4-octyl itaconate (4-OI) (125 μM) and collected after an 8 Gy-radiation for 4 h or 24 h. **(H)** Representative expression of NRF2 in H1299 treated with NAC and/or 4-OI (125 μM) after an 8Gy-radiation detected by western blot. **(I)** mRNA expression level of *ATF3* in H1299 cells transfected with NC or different siRNAs. **(J)** Representative images and colony number per well of H1299 cells transfected with NC or siRNA and treated with DMSO or 4-octyl itaconate (4-OI) (125 μM) after radiation. **(K)** Representative images and colony number per well of H1299 cells treated with 4-octyl itaconate (4-OI) (125 μM) or C-176 (5 μM) after radiation. *p < 0.05; **p < 0.01; ***p < 0.001; ns, not statistically significant.

**
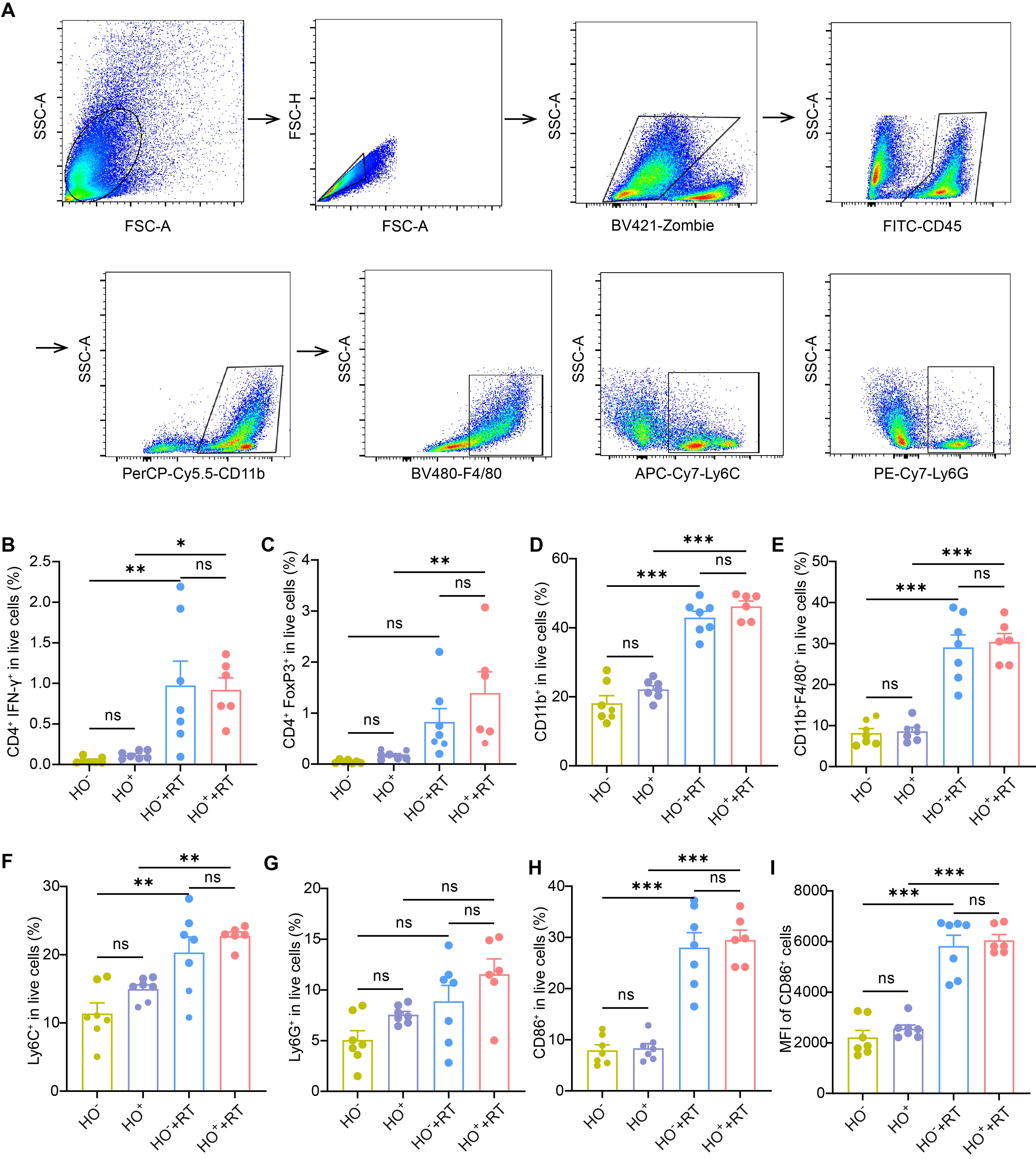
Fig S5: The immune cells in the TME.**

**(A)** Gating strategy for detection of macrophages and MDSCs by flow cytometry. **(B-G)** Flow cytometry analysis of CD4^+^ IFNγ^+^ **(B)**, CD4^+^ FoxP3^+^ **(C)** T cells, CD11b^+^ myeloid cells **(D)**, CD11b^+^ F4/80^+^ macrophages **(E)**, and Ly6C^+^ MDSCs **(F)** in the TME of LLC subcutaneous tumor model in mice treated with radiation (8 Gy x 3) in *Acod1^f/f^ Lyz2^cre--^* (HO^-^) and *Acod1^f/f^ Lyz2^cre+^* (HO^+^) mice (n = 6). **(H-I)** The percentage of CD86^+^ **(H)** and MFI of CD86 **(I)** in the TME of LLC subcutaneous tumor model (n = 6). *p < 0.05; **p < 0.01; ***p < 0.001; ns, not statistically significant.

**Supplementary Table 1. Sequences of siRNAs in this study.**

| **Name** | **Sequence** |
| --- | --- |
| *RELA-1 SenseSeq5‘→3’* | GGACAUAUGAGACCUUCAAGATT |
| *RELA-1 AntiSeq* | UCUUGAAGGUCUCAUAUGUCCTT |
| *RELA-2 SenseSeq5‘→3’* | GGCGAGAGGAGCACAGAUACCTT |
| *RELA-2 AntiSeq* | GGUAUCUGUGCUCCUCUCGCCTT |
| *RELA-3 SenseSeq5‘→3’* | GAGUCAGAUCAGCUCCUAATT |
| *RELA-3 AntiSeq* | UUAGGAGCUGAUCUGACUCTT |
| *NFE2L2-1 target seq* | GAGAAAGAATTGCCTGTAA |
| *NFE2L2-2 target seq* | GCTACGTGATGAAGATGGA |
| *NFE2L2-3 target seq* | GCCCTCACCTGCTACTTTA |
| *ATF3-1 SenseSeq5‘→3’* | CCUGAAGAAGAUGAAAGGAAATT |
| *ATF3-1 AntiSeq* | UUUCCUUUCAUCUUCUUCAGGTT |
| *ATF3-2 SenseSeq5‘→3’* | GCAUUUGAUAUACAUGCUCAATT |
| *ATF3-2 AntiSeq* | UUGAGCAUGUAUAUCAAAUGCTT |
| *ATF3-3 SenseSeq5‘→3’* | CCUCUUUAUCCAACAGAUAAATT |
| *ATF3-3 AntiSeq* | UUUAUCUGUUGGAUAAAGAGGTT |

**Supplementary Table 2. Sequences of primers for qPCR.**

| **Primer Name** | **Primer Sequence** |
| --- | --- |
| *Rps18* Forward | CGGAAAATAGCCTTCGCCATCAC |
| *Rps18* Reverse | ATCACTCGCTCCACCTCATCCT |
| *Irg1* Forward | AGTTTTCTGGCCTCGACCTG |
| *Irg1* Reverse | AGAGGGAGGGTGGAATCTCT |
| *ACTB* Forward | CTCACCATGGATGATGATATCGC |
| *ACTB* Reverse | AGGAATCCTTCTGACCCATGC |
| *NRF2* Forward | CACATCCAGTCAGAAACCAGTGG |
| *NRF2* Reverse | GGAATGTCTGCGCCAAAAGCTG |
| *NQO1* Forward | CCTGCCATTCTGAAAGGCTGGT |
| *NQO1* Reverse | GTGGTGATGGAAAGCACTGCCT |
| *GCLM* Forward | TCTTGCCTCCTGCTGTGTGATG |
| *GCLM* Reverse | TTGGAAACTTGCTTCAGAAAGCAG |
| *HMOX1* Forward | CCAGGCAGAGAATGCTGAGTTC |
| *HMOX1* Reverse | AAGACTGGGCTCTCCTTGTTGC |
| *GSR* Forward | TATGTGAGCCGCCTGAATGCCA |
| *GSR* Reverse | CACTGACCTCTATTGTGGGCTTG |
| *RELA* Forward | AGAGCAGCGTGGGGACTAC |
| *RELA* Reverse | ATGGGATGAGAAAGGACAG |
| *ATF3* Forward | CGCTGGAATCAGTCACTGTCAG |
| *ATF3* Reverse | CTTGTTTCGGCACTTTGCAGCTG |

**Supplementary Table 3. Antibodies mentioned in the study.**

| **Antibodies** | **Source** | **Detail information** |
| --- | --- | --- |
| γH2AX | Cell Signaling Technology | #2577, 1:500 for IF |
| CD8 | Abcam | #ab237364,1:500 for IF |
| Acod1 | Abcam | #ab238580,1:200 for IF |
| CD68 | Proteintech | #66231-2-Ig,1:2000 for IF |
| Acod1 | Abcam | #ab222411,1:1000 for WB |
| P65 | Proteintech | #10745-1-AP, for ChIP |
| P65 | Proteintech | #10745-1-AP, 1:2000 for WB |
| LC3 A/B | Servicebio | GB11124-50, 1:1000 for WB |
| P62 | Proteintech | #18420-1-AP, 1:5000 for WB |
| p-P65 | Cell Signaling Technology | #3033, 1:1000 for WB |
| NRF2 | Proteintech | #16396-1-AP, 1:2000 for WB |
| β-Actin | Proteintech | #66009-1-Ig,1:20000 for WB |

**Supplementary Table 4. Sequences of primers for ChIP-qPCR**

| **Primer Name** | **Primer Sequence** |
| --- | --- |
| *Irg1* Forward | AATGGGCTGTCTGTGAGA |
| *Irg1* Reverse | CCAGGTCTGTCCCTTTCAC |
